# Predictors of Continued Breastfeeding at One Year among Women Attending Primary Healthcare Centers in Qatar: A Cross-Sectional Study

**DOI:** 10.3390/nu10080983

**Published:** 2018-07-27

**Authors:** Amal Nasser, Fadumo Omer, Fatima Al-Lenqawi, Rehab Al-awwa, Tamam Khan, Asmaa El-Heneidy, Rana kurdi, Ghadir Al-Jayyousi

**Affiliations:** Department of Public Health, College of Health Sciences, Qatar University, 2713 Doha, Qatar; an1205803@student.qu.edu.qa (A.N.); fo1203580@student.qu.edu.qa (F.O.); fa1203503@student.qu.edu.qa (F.A.-L.); ra1208955@student.qu.edu.qa (R.A.-a.); tk1202457@student.qu.edu.qa (T.K.); mph.asmaa@gmail.com (A.E.-H.); rana.kurdi@qu.edu.qa (R.k.)

**Keywords:** breastfeeding, knowledge, practice, barriers, social support, professional support

## Abstract

The number of babies in Qatar being exclusively breastfed is significantly lower than the global target set by the World Health Organization. The purpose of this study was to assess knowledge, attitude, and practice (KAP), selected barriers, and professional support as well as their association with continued breastfeeding at one year of age. A sample of Qatari and non-Qatari mothers (*N* = 195) who attended a well-baby clinic held at primary health care centers in Qatar completed a self-administered questionnaire. Descriptive analysis, the Pearson Chi-squared test, and logistic regression were performed. Around 42% of the mothers stopped breastfeeding when their child was aged between 0 and 11 months old. Mothers who had only one or female child stopped breastfeeding between the ages of 0 and 6 months (*p* = 0.025, 0.059). The more optimal the breastfeeding practices followed by the mothers, the older the age of the infant when they stopped breastfeeding (*p* = 0.001). The following factors were inversely associated with breastfeeding duration: the mother’s perceptions that she “did not know how to breastfeed,” or “wasn’t making enough milk,” and the need “to return to work/school”, with *p* = 0.022, 0.004, and 0.022, respectively. These findings present factors that should be considered when planning for health education and promotion programs to prolong breastfeeding duration in Qatar.

## 1. Introduction

Exclusive breastfeeding contributes greatly to providing better health outcomes by preventing disease and promoting health in both the short and long term for mothers and their children [1]. It also reduces infant mortality from common childhood illnesses. Approximately 800,000 children’s lives could be saved globally each year if every child was exclusively breastfed for the first six months of life [2].

Despite the established importance of breastfeeding, in the Middle East and North Africa (MENA), regional fact sheet-recommended levels are still not attained. The rate of exclusive breastfeeding continuation in Qatar is 12%, which is low when compared with the global rate of 37%. Qatar’s rate is also significantly lower than the global target set by the World Health Organization, which calls for at least 50% of babies under six months of age to be exclusively breastfed by 2025 [3].

Mothers who do not breastfeed their infants and depend on formula milk may suffer from increased incidence of premenopausal breast cancer, ovarian cancer, retained gestational weight gain, type 2 diabetes, myocardial infarction, and metabolic syndrome. Additionally, infants who are not breastfed are more likely to suffer from infectious morbidity, childhood obesity, diabetes (types 1 and 2), leukemia, and sudden infant death syndrome [4].

Qatar ranks 10th in the world and third in the MENA region for diabetes. The nation has a comparative prevalence of diabetes of 22.9% and an estimated 283,000 diabetes patients between 20 and 75 years of age [5]. Therefore, understanding the factors affecting breastfeeding and how to enhance and prolong the duration of breastfeeding is vital for diabetes prevention in the region.

### 1.1. Continued Breastfeeding at One Year: A Core Indicator to Assess Breastfeeding Practices

Globally in 2015, the percentage of children with continued breastfeeding at one year of age was 74% [6]. Regionally, the proportion of children with continued breastfeeding up to one year in Oman was reported to be 95% in 2000. This is much higher than the rate of Qatar, where it was 4% among Qataris and 69.6% among non-Qataris (non-Qataris refer to any immigrant in Qatar—not only limited to Middle Eastern immigrants, but also Arabs from the Gulf Cooperation Council (GCC) region, other Arab regions, and non-Arab nationalities [7]). The World Health Organization uses core indicators to assess breastfeeding practices among women [8]. Since the majority of mothers in Qatar stop breastfeeding within one year, it is crucial to study the “continued breastfeeding at one year” indicator, which is defined as the proportion of children aged 12–15 months who are fed breast milk.

### 1.2. Factors Influencing Breastfeeding Continuation at One Year

Qatar is a rapidly developing country with massive reserves of oil and natural gas. Globally, it is considered to have the second-largest natural gas reserves. It is part of the GCC region and has a population of 2,700,000, which includes only 320,000 Qatari citizens. The remaining are residents from a large range of nationalities and comprise the main work force in the country.

Previous research has found that nationality, education, and financial status are among the factors that influence breastfeeding duration. Al-Darweesh and colleagues found that non-Kuwaiti women with postgraduate degrees practiced breastfeeding longer than other Kuwaiti women [9,10] and that rich families left traditional practices and used formula feeds [11].

Knowledge, attitude, and practice (KAP) of breastfeeding also contribute to continued breastfeeding at one year of age. In GCC countries, research found that breastfeeding knowledge among studied participants was highly adequate [12]. However, continued breastfeeding practices at one year were not sufficient, and was negatively influenced by mother’s attitudes toward breastfeeding [13]. In addition, some breastfeeding practices have contributed to continued breastfeeding at one year of age, including mother–infant skin-to-skin contact (SSC) after birth, which was shown as an efficient method in supporting the continuation of breastfeeding [14]. However, pacifier usage and using only breast milk to feed infants six months and older without complementary foods were factors that were found to promote weaning [15,16].

Factors that are considered as barriers to continued breastfeeding include mothers’ perception of pain, body image, body changes, embarrassment from breastfeeding in public, and breastfeeding at work. Barriers arising from social norms are also documented in the literature: some mothers subscribe to the norm “bigger is better,” which means fat babies are healthier. This leads them to use bottled milk and introduce solid food earlier than recommended [9,11,17].

Social support provided by family members also shapes continued breastfeeding at one year of age. Women rely on support and advice from their mothers and grandmothers, who may provide practical support to continue breastfeeding for up to two years. Research has reported that support from a spouse was also positively associated with longer duration of breastfeeding [17,18]. With regards to professional support, healthcare professionals can also provide a vital role for breastfeeding mothers. Face-to-face pre-natal and postnatal classes proved to be effective in reducing early cessation of breastfeeding and promoted breastfeeding prevalence [19].

The unique context in Qatar causes more challenges to continued breastfeeding than in other GCC countries. A previous study reported that mothers in Saudi Arabia than Qatar are more open to breastfeeding in front of the family and in public [11]. Relatively little is known about breastfeeding continuation practices up to one year of age among mothers in Qatar. The purpose of this research is to study demographics, assess KAP regarding breastfeeding, explore barriers to breastfeeding, and study selected aspects of professional support and their association with continued breastfeeding at one year of age among mothers visiting primary healthcare centers in Qatar.

## 2. Materials and Methods

A cross-sectional study was conducted between February and June 2017. Participants were randomly selected mothers attending well-baby clinics at five primary healthcare centers in Doha, Qatar. The study locations were selected based on geographical distribution; they were also training sites for the first five authors.

A selection of Qatari and non-Qatari mothers who attended well-baby clinics was chosen. The target sample was 200 with 40 mothers per center. To be eligible for the study, mothers had to have given birth to their youngest baby in Qatar (participant baby), lived in Qatar for at least two-thirds of the pregnancy, and attended a well-baby clinic at one of the chosen centers. A total of 195 questionnaires were completed by participants.

The study was approved by the Qatar University Institutional Review Board (Research Ethics Approval No is QU-IRB 387-E/17) and was conducted in full agreement with the rules and regulations of the Primary Healthcare Care Cooperation (PHCC) Research Section. A consent form was read and signed by participants who agreed to participate prior to filling out the questionnaire.

### 2.1. Data Collection

Data were collected anonymously using a self-administered questionnaire, which was prepared in English and Arabic. Participants were randomly selected from the different five centers. We approached mothers in the well-baby clinic and briefly explained the purpose of the study. Mothers who agreed to participate were handed a consent form to sign prior to completing the questionnaire. A cover letter was attached to each questionnaire, addressing the purpose of the study and emphasizing confidentiality.

The estimated time for completion of the survey was 15–20 min. The opportunity to ask any question was given to the participants. Mothers completed the questionnaire and returned it to the data collectors. Participation was voluntary, and mothers were able to refuse any question they did not wish to respond to.

### 2.2. Overview of Survey

An extensive literature review of studies on breastfeeding was conducted to identify potential items of the study instrument. Based on the literature review, the questionnaire was adapted from validated surveys that have been used locally, regionally, and globally. It was translated into Arabic and subjected to a process of forward and backward translation by a research group at Qatar University. It was pretested on 20 mothers who attended well-baby clinics at primary health care institutions in Doha, and modifications were made as necessary so that it was clear and would provide accurate information.

The first section of the questionnaire related to socio-demographic characteristics of the participants, such as age, nationality, religion, education level for mothers and husbands, occupation, living with a husband, total monthly income, number of children, mode of delivery, last age of child, sex of child, and order. These questions were adapted from previous studies conducted in Saudi Arabia and Iran [9,10,17,19,20]. The second section covered breastfeeding knowledge, attitude, and practice. The breastfeeding knowledge part was used to assess the participants’ knowledge of four aspects: benefits to the baby (it provides more protection from allergies compared to formula milk and reduces the risk of childhood obesity and chronic diseases such as diabetes), benefits to mothers (exclusive breastfeeding is beneficial in spacing births, achieving pre-pregnancy weight faster, and leads to a lower risk of developing breast and ovarian cancer), duration of breastfeeding, and effective feeding (breastfeeding should initiate immediately after delivery, infants should be exclusively breastfed for the first 6 months, breastfeeding is recommended up to one year of age, and the optimal age to stop breastfeeding is two years of age). These questions were adapted from previous research in Kuwait and Saudi Arabia [21,22]. For benefits to baby statements, a five-point Likert scale (1 = strongly agree and somewhat agree, 0 = somewhat disagree, strongly disagree, and neutral) was used to evaluate the participants’ responses. For the benefits to mothers, duration of breastfeeding, and effectiveness of breastfeeding, items had several categories of responses (yes, no, or do not know).

The part on breastfeeding attitudes focused on reasons behind the adoption of breastfeeding, and items had response categories of yes, no, and do not know [21,22]. The breastfeeding practice questions asked about the number of children who were breastfed, the starting time of breastfeeding, the age of the child when mothers introduced formula or other milk, the practice of skin-to-skin contact, amount of water given to the baby after breastfeeding, and pacifier usage after delivery [7,9,14,21,23].

In the third section, participants were asked whether they had stopped breastfeeding, the age of the child when they completely stopped breastfeeding, and reasons that made women decide to stop breastfeeding. These questions were adapted from different studies [21,22]. Furthermore, there was a question reflecting social support (“Who had the most impact on you to continue breastfeeding?”) and participants were able to select more than one answer. The choices included mother, partner, mother-in-law, and friends [21].

Section four was about professional support provided by healthcare providers for breastfeeding mothers. This section included questions about whether mothers received training about proper positioning during breastfeeding and support for feeding problems after delivery, if the importance of breast milk was explained by a physician, if they received breastfeeding health education, and if the hospital provided them with breastfeeding tools [16,19,22]. This section included five questions and items had two categories of responses (yes and no). At the end of the survey, an open-ended question was used to elicit the participants’ opinion on how professionals can support mothers for prolonged breastfeeding.

### 2.3. Statistical Analysis

Data generated by the questionnaire were appropriately coded and analyzed using Software Package for Social Sciences (SPSS) version 23. Regarding the breastfeeding knowledge part, a correct response was scored as 1, while a wrong answer/“do not know” response was scored as 0. The knowledge score was calculated by adding the correct responses to 14 statements and categorized into three levels, which were poor (0–4), moderate (5–9), and good (10–14).

The first and second statements in the breastfeeding attitude part (“The community prefers breastfeeding over artificial feeding” and “Breastfeeding reduces family expenses”) were scored as “yes” = 1, “no”, “do not know” = 0. The third statement (“It is difficult for a breast feeder to take care of her family”) was scored as “yes” = 1, “no”, “do not know” = 0. To calculate the total attitude score, we classified those that answered two or three statements correctly as having a positive attitude, while others who answered zero or one statement correctly as having a negative attitude.

The breastfeeding practice part included nine questions. Out of these questions, five featured “yes” and “no”, options—the correct answer was scored as 1 while a wrong answer was scored as 0. For the question “How many of your children did you breastfeed?”, the response “all or some of them” was scored as 1, and the response “only the first child only or last child only” was scored as 0. For the question “When did you start breastfeeding?”, the answer was scored as 1 if they answered that it had occurred within 1 or 6 h of delivery and was scored as 0 if they responded that it had occurred after 6 h of delivery, after 24 h of delivery, or that they did not breastfeed. The overall practice score was categorized into two levels, indicated as non-optimal (0–4) and optimal (5–9). Optimal practice means that mothers breastfed all or some of their children, practiced skin-to-skin contact, practiced breastfeeding within 1 or 6 h of delivery, did not introduce formula or other milk before 6 months of age, did not give water to the infant after every breastfeed, and did not use a pacifier after delivery.

In the third section, the answers to the question “Did you stop breastfeeding?” were scored as “yes” = 1 and “no” = 0. For the question “How old was your infant when you totally stopped breastfeeding?”, the answers were scored as “Have not stopped” = 0, “0–6 months” = 1, “7–11 months” = 2, and “12 months or more” = 3. Questions about the reasons that made women decide to stop breastfeeding included nine statements. We scored the participants’ responses as “not at all” = 0, and “a little”, “somewhat”, and “a lot” = 1. The last section, regarding professional support, included five questions for which the possible responses were “yes” and “no”. A correct response was scored as 1 while a wrong response was scored as 0. Lastly, a thematic analysis was conducted for participants’ responses to the open-ended question to understand the mothers’ recommendations on how to prolong the duration of breastfeeding.

Descriptive analysis was performed for mothers’ demographics, breastfeeding knowledge, attitude and practice, barriers, and professional support. The bivariate Chi-squared analysis was used to describe the categorical variables and study the association with outcome 1 “Did the mother stop breastfeeding?” and outcome 2 “Infant age when breastfeeding was stopped”. A bar chart was used for breastfeeding adoption question, barriers, and professional support to show the comparison of each category in a frequency distribution. The significant variables from the Chi-squared test were used to fit a model about the possible predictors of outcomes one and two by performing multivariate logistic regression, *p*-values less than 0.05 were considered to be significant.

## 3. Results

The majority of the participants in this study were aged between 31 and 35 years (31.8%), were non-Qatari (around 60%), Muslim (87.7%), and had university education or higher (74.4%). In terms of the mother’s occupation, 53.3% of the mothers were housewives and most of the participants (64.1%) had a monthly income between 0–20,000 QR ($5500) (Table 1). The majority of the participant babies were aged 12 months or older (57.7%), around 29.2% were aged 0–6 months, and 12.8% of the babies were aged 7–11 months.

### 3.1. Descriptive Analysis

Our study showed that overall breastfeeding knowledge based on four aspects (the benefits to the baby, the benefits to mothers, the duration of breastfeeding, and effective feeding) was possessed by 80% of the mothers.

**Figure 1:**
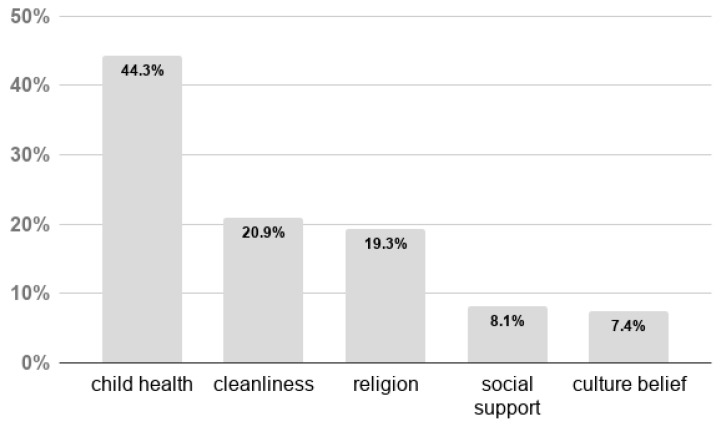
Reasons behind the adoption of breastfeeding among mothers attending the Primary Healthcare Care Cooperation (PHCC).

The majority of the mothers (89%) reported a positive attitude towards breastfeeding. Most of the respondents agreed that “The community prefers breastfeeding over artificial feeding” (78.9%), and “Breastfeeding reduces family expenses” (82.5%). Around 76% of the mothers believed that breastfeeding did not affect taking care of the family when presented with the statement: “It is difficult for the breastfeeding mother to take care of her family.” They also reported that the major reasons behind the adoption of breastfeeding were child health (44.3%), followed by cleanliness (20.9%), and religious reasons (19.3%). The latter is related to Islamic beliefs stated in the Holy Quran that “…mothers shall breastfeed their children for two whole years for those who desire to complete the appropriate duration of breastfeeding” (2:223) (Figure 1).

Regarding breastfeeding practices, around 94% of participants breastfed their children, and the majority (78.8%) initiated breastfeeding within the first 6 h of delivery (Table 2). Yet, around 78% of participants gave their children formula or other milk and most of them started this between the first and third month of the infant’s life (40.6%). This may have influenced the exclusive breastfeeding rates among the participants at 1–3 months, 4–6 months, and after 6 months, which were 40%, 20%, and 10%, respectively.

**Figure 2:**
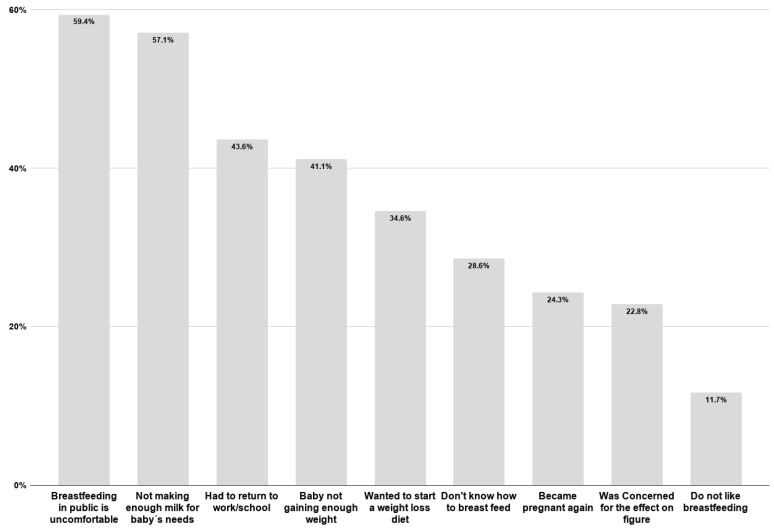
Barriers to continued breastfeeding as identified by mothers attending the PHCC.

Table 3 shows that, among mothers who had already stopped breastfeeding (76.8%), 42% had stopped before the age of 12 months. Participants explained that major factors prohibited them from continuing to breastfeed their infants. These reasons include being uncomfortable with breastfeeding in public (59.4%), not producing enough milk for their baby’s needs (57%) and having to go back to work or school (43.6%) (Figure 2).

However, participants reported that mothers had the most influence on their continuation of breastfeeding (52.90%), followed by partners and mothers-in-law (17.60%). Regarding professional support provided by healthcare providers at PHCCs, participants were mostly satisfied with the physicians’ explanation regarding the importance of breastmilk, followed by breastfeeding health education messages provided during or after pregnancy. Only half of mothers received training about the proper position during breastfeeding and thought that there was enough support for feeding problems after delivery. Even though mothers received knowledge and information regarding the importance of breastfeeding, they were not provided the crucial skills needed to follow an effective feeding process and manage any feeding issue faced after delivery.

### 3.2. Bivariate Analysis

Table 4 presents the association of selected demographics of the participants, KAP, and barriers of continued breastfeeding with outcome 1 (if the mother stopped breastfeeding) and outcome 2 (infant age when stopped breastfeeding). The Chi-squared test showed that 81.3% of women who stopped breastfeeding were aged between 21 to 25 years old and, according to outcome 2 (infant age when stopped breastfeeding), 50% of these women stopped breastfeeding early (0–6 months of age). Qatari mothers who stopped breastfeeding when their infant was between 0 to 6 months of age represented 41.9% of the studied population. However, 36.8% of non-Qataris mothers continued breastfeeding to 12 months, which was longer compared to the Qataris mothers (31.1%).

The work environment was a major barrier to continued breastfeeding, as the majority of employed mothers (80.2%) reported that they had stopped breastfeeding. Additionally, 40.7% of employed mothers stopped breastfeeding when their infants were between 0 and 6 months of age, compared to 30% of housewives. With regard to family income, 43.3% of mothers who had a high income (more than 20,000 QR, approximately $5500) reported that they stopped breastfeeding when the infant was between 0 and 6 months of age. A higher proportion of mothers (38.1%) with a lower income stopped breastfeeding their infant before 12 months as compared to mothers with a higher income (29.9%).

The results showed that 84% of participants with two children had stopped breastfeeding. There was a significant association (*p* = 0.025) between the number of children and outcome 2 (infant age when BF was stopped); 49% of mothers with only one child stopped breastfeeding within the first 6 months and they were the least likely to continue breastfeeding their infants to 12 months or more (20.4%). The sex of the child could also play an important role in continued breastfeeding up to one year of age. Our results showed that 80.4% of mothers stopped breastfeeding their female child earlier than their male child. There was a slight significant association between the child’s sex and outcome 2 (*p*-value = 0.059). In addition, 44.3% of mothers stopped breastfeeding their female infants between the ages of 0 and 6 months, while 40.5% of mothers reported that their infant males received breast milk for up to 12 months.

The results showed that mothers tended to stop breastfeeding despite good breastfeeding knowledge because of the influence of other individual, sociocultural, and environmental factors. The majority of women who had a negative attitude toward breastfeeding (42.1%) and disclosed non-optimal breastfeeding practices (87.9%) stated that they stopped breastfeeding between 0 and 6 months. Among mothers who had optimal practice, 26.5% of them were still breastfeeding their babies (*p* = 0.001) and (38.6%) of these mothers breastfed their children for 12 months or more.

The analysis showed that around 83% of the mothers stated that they stopped breastfeeding because they did not know how to breastfeed, and there was a statistically significant relationship between this barrier and outcome 2 (*p* = 0.022). Around 53% of mothers stopped breastfeeding their infant between the ages of 0 and 6 months because they did not know how to do it. Not making enough milk for the baby’s needs was a barrier that many mothers faced. Around 78% of the respondents reported that they stopped breastfeeding due to this barrier and 43.6% of those stopped breastfeeding their infant between 0 and 6 months of age. There was a significant relationship between this barrier and outcome 2 (*p* = 0.001).

Considering the work environment, which acts as a huge barrier to breastfeeding for employed mothers, our results showed that there is a significant relationship between going back to work and outcome 1 (whether the mother had stopped breastfeeding) (*p* = 0.022). Around 86% of the respondents reported that they had stopped breastfeeding because they had to go back to work and 43.4% of those stopped breastfeeding between 0 and 6 months of age. Other barriers to continued breastfeeding were “disliking breastfeeding”, preferring other methods (such as bottled milk), and mother’s concerns about her figure. However, none of these factors showed a statistically significant relationship with the outcomes.

### 3.3. Multivariate Logistic Regression of Barriers by Outcome 1

The significant variables from the Chi-squared test were used to fit a model about the possible predictors of outcome 1 (whether the mother had stopped breastfeeding). The findings show that “Had to return to work/school” was the only significant predictor (*p* = 0.021), (odds ratio (OR) 2.698, 95% confidence interval (CI) 1.162, 6.267). Therefore, mothers who had to go back to work were more likely to stop breastfeeding because of the work environment was prohibitive to practicing breastfeeding (see Table 5).

### 3.4. Qualitative Analysis

A thematic analysis was conducted for participants’ responses to an open-ended question about their recommendations to prolong breastfeeding duration. Most of the participants thought that raising awareness about the importance and benefits of breastfeeding for the mother and her child was necessary. This applied especially to new mothers and one of the participants suggested the provision of “…awareness lectures for mothers before and after giving birth about the importance of breastfeeding.”

Many mothers suggested having professional support aimed at providing training courses/practical sessions for new mothers about the appropriate positions to breastfeed and providing health advice about suitable nutritional intake for mothers during breastfeeding. One of the participants stated that health professionals should: “explain to mothers the importance of skin-to-skin contact to increase the quality and quantity of the breast milk and to not use formula milk for the baby during the first months.”

Finally, one of the recommendations was to have longer maternity leave, since working mothers face difficulties in balancing work and breastfeeding. Some of the participants mentioned that “Maternity leave is only 60 days, which is not enough to give baby breast milk” while another suggested extending “…maternal leave to 4–6 months so the mother will not stop breastfeeding and will not leave her child for long hours.”

## 4. Discussion

The present study assessed selected factors and their association with continued breastfeeding at one year of age among mothers attending well-baby clinics at five primary health care in Qatar. Around 77% of the participants in our study reported that they had stopped breastfeeding and 42% of those had stopped within the infant’s first year of life. Mothers in our study were more likely to stop breastfeeding early (0–6 months of infant’s age) if they were between 21 to 25 years of age, had only one infant (first-time mothers), were employed, and had high incomes (more than 20,000 QR). Similar findings were reported in Saudi Arabia, where it was found that increased maternal age, lower levels of education, and low income contributed to longer duration of breastfeeding [20]. Therefore, it is crucial that these young women are targeted for breastfeeding educational campaigns to enhance their awareness of the importance of breastfeeding for them and their infants [23].

Culture and social norms play a role in influencing the duration of breastfeeding depending on the sex of the child. Our results showed that male infants were more likely to be breastfed for up to 12 months than female infants. This aligns with findings from Turkey, which showed that male infants are breastfed for one or two months longer than females [24]. In an Arab culture, mothers are always pleased if they have a baby boy and they do their best to raise a healthy man. As a result, these cultural values shape their breastfeeding practices and lead them to spend more time breastfeeding their male infants than females. Addressing religion when communicating to mothers, indicating that Islam encourages mothers to breastfeed their infants for two years without preference for either sex, could help promote the duration of breastfeeding for both sexes.

At the individual level, we found that most of the participating mothers have adequate breastfeeding knowledge and a positive attitude. However, almost half of them stopped breastfeeding their infants before one year of age. These results are consistent with previous research in which having a high breastfeeding knowledge level did not enhance the rate of continued breastfeeding [25]. Other factors should thus be addressed to help explain why mothers stop breastfeeding, despite being aware of the benefits to them and their infants. Assessing practices followed by participants and other social and environmental factors could help explain what prohibits these mothers from continuing breastfeeding until the child is one year of age.

Participants reported following a variety of breastfeeding practices. Those who disclosed that they had good practices were more likely to breastfeed their children for 12 months or more, while others who had bad practices stopped breastfeeding after 0–6 months. Most of those who disclosed bad practices gave their infants formula or other milk between the first and the third month. Some mothers gave water to the child after every breastfeed and around half of the mothers used a pacifier after delivery, which may have accelerated the weaning process. Similar findings have been reported by previous research [26]. As a consequence, in our study only 10% of the participants exclusively breastfed their infants for the first six months, which is considered a low rate according to the WHO recommendations, but higher than exclusive breastfeeding rates from other regions. Kamudoni and colleagues (2007) reported that exclusive breastfeeding rates in Malawi at 2, 4, and 6 months of age were 39.1%, 27.5%, and 7.5%, respectively [27]. An explanation for these findings could be the lack of antenatal breastfeeding education and postnatal support provided by healthcare providers, which was strongly recommended by participants to enhance the duration of breastfeeding. Breastfeeding educators must contribute effectively to the promotion of breastfeeding and alleviate the current gap between knowledge and breastfeeding practice, so that it can be re-incorporated into the lives of Qatari women [11].

Other significant barriers to continued breastfeeding reported by participants were “not making enough milk for baby’s needs” and “did not know how to breastfeed.” These mothers were more likely to stop breastfeeding early, in the first 6 months. Our findings are consistent with previous research which showed that if women felt that their babies were not satisfied with breastfeeding, it might negatively influence breastfeeding duration [28]. Many women utilize infant satisfaction cues as their main indication of milk supply, since actual milk supply is difficult to measure [29,30]. A recent study conducted by Galipeau et al. has concluded that mother’s perception of not having sufficient milk was not associated with an actual insufficiency of milk supply, as measured by the baby’s weight loss or 24-h milk production. The authors found that factors associated with maternal perception versus actual insufficiency were different and therefore they recommended that interventions should be directed toward promoting early, optimal, and frequent feedings [31]. Training sessions about appropriate breastfeeding positions and practices to increase breastmilk should be incorporated into breastfeeding promotion campaigns. Another barrier to continued breastfeeding reported by the participants was feeling that “breastfeeding in public is uncomfortable.” A study conducted in Saudi Arabia found that the chance of stopping breastfeeding increased among mothers who perceived lactation in public as a barrier [32].

In Qatar, one study found that women who practiced breastfeeding because they were aware of the benefits for them and their infants still struggle to commit to practice due to limited social support. Family has been recognized as a crucial context to promote breastfeeding duration by providing social support to mothers. It has been reported that mothers and mothers-in-law provide social support and encourage breastfeeding mothers to continue to one year of age [28]. These findings align with those of our study, which showed that mothers and mothers-in-law of the participants were the biggest providers of social support to continue breastfeeding for one year. In addition, partners provided less support. This indicates that various family members should be included in health education and promotion programs that aim to enhance the duration of breastfeeding among mothers in Qatar.

Multiple logistic regressions in our study showed that the most significant predictor of continued breastfeeding to one year was returning back to work or school. Mothers reported that if they had to go back to work, then they were more likely to stop breastfeeding their infants, which suggests that these women had difficulty combining work with breastfeeding. A Kuwaiti study disclosed work as a barrier to continued breastfeeding, with women reporting that they could not breastfeed and work. The Department of Health and Human Services in the US has claimed that returning to work is a barrier to continued breastfeeding, since mothers usually have less time and do not have an appropriate place to breastfeed at work. The creation of breastfeeding-conductive spaces in public and at work to increase social acceptability of breastfeeding and, as a consequence, enhance the duration of breastfeeding has been recognized by new initiatives in Gulf Cooperation Council (GCC) countries [30]. Additionally, participants in our study recommended increasing maternity leave to six months, which will enable them to spend more time with their infants and, therefore, enhance the probability of continued breastfeeding to one year.

Limitations in this study include the cross-sectional design, which limits the ability to infer the causation between the various factors and continued breastfeeding at one year. Using a self-administrated survey may lead to recall bias. The small sample size (this was a pilot study) limits the generalization to all mothers attending well-baby clinics at PHCCs in Qatar.

The current study provides baseline information and addresses a gap in the literature regarding factors influencing continued breastfeeding among mothers attending primary health care in Qatar. Understanding these factors will provide evidence for policy makers and breastfeeding educators to plan for effective health promotion programs that promote breastfeeding practices and prolong the duration, which align with the major goals of the national public health strategy in Qatar [33]. Improving the health and well-being of mothers and their infants will decrease the prevalence of major health issues the country is facing, including obesity and diabetes. Future research should focus on studying the factors and determinants associated with continued breastfeeding using a large, representative sample of mothers attending PHCCs in Qatar. More research is also needed to study actual breastfeeding practices in the country in relation to international recommendations.

## 5. Conclusions

Our study is the first of its kind in Qatar, assessing different factors on different levels and their association with continued breastfeeding at one year of age. Around 42% of the participants in our study had stopped breastfeeding within one year and only 10% of them did not use formula or other milk before six months of age. This is reflected in the low rate of exclusive breastfeeding in our sample. The findings suggested that continuation of breastfeeding is a process influenced by a variety of individual, sociocultural, and environmental factors which should be considered when planning for effective interventions to enhance continued breastfeeding until one year of age. First-time mothers, Qataris, and high-income mothers were more likely to stop breastfeeding when their child was between 0 and 6 months old. Working mothers and mothers who did not know how to effectively breastfeed their infants were also more likely to stop breastfeeding within one year.

Implementing the WHO and UNICEF (United Nations International Children’s Emergency Fund) recommendations for breastfeeding-friendly hospitals in Qatar will improve continued breastfeeding to one year and beyond. Community-based interventions can facilitate the implementation of these recommendations and enhance exclusive and continued breastfeeding until one year of age. Breastfeeding educators and other healthcare providers can work with mothers to provide the skills needed for optimal breastfeeding practices, including appropriate breastfeeding positioning and practices that increase breastmilk. Educators can help mothers to seek family members for social support to enhance continued breastfeeding for at least one year and can help policy makers advocate for friendly breastfeeding work environments and longer maternity leave.

## Figures and Tables

**Table 1:** Socio-demographic characteristics of participants.

Characteristics	*N* (%)
Age	
21–25	36 (18.5%)
26–30	53 (27.2%)
31–35	62 (31.8%)
36–40	44 (22.6%)
Nationality	
Qatari	79 (40.50%)
Non-Qatari	115 (59.0%)
Religion	
Muslim	171 (87.7%)
Non-Muslim	24 (12.3%)
Mother’s education	
High school or less	50 (26.6%)
University education or higher	145 (74.4%)
Mother’s occupation	
Housewife	98 (53.3%)
Employee	86 (46.7%)
Income	
0–20,000 ($5500)	123 (64.1%)
More than 20,000	69 (35.9%)
Smoking	
Yes	3 (1.5%)
Number of children	
One	58 (29.7%)
Two	52 (26.7%)
Three or more	85 (43.6%)
Delivery mode	
Normal delivery	122 (62.6%)
Caesarean section	73 (37.4)
Sex of last child	
Male	91 (46.7%)
Female	104 (53.3%)

**Table 2:** Breastfeeding practices by mothers attending primary healthcare centers

Statements (Variables)	*N* (%)
Had breastfed a child	182 (93.8%)
Number of children breastfed	
First child only or last child only	53 (28.2%)
All of them or some of them	135 (71.8%)
Practiced skin-to-skin contact	162 (85.7%)
Timepoint when breastfeeding started	
Within 1 or 6 h of delivery	152 (78.8%)
Did not breastfeed, or breastfed after 6 h but within 24 h of delivery	41 (21.2%)
Used formula or other milk	48 (77.5%)
Baby’s age when the formula or other milk was introduced	
Less than 1 month	47 (29.4%)
1–3 months	65 (40.6%)
4–6 months	32 (20.0%)
More than 6 months	16 (10.0%)
Giving water to the baby is encouraged after every breastfeeding	72 (38.3%)
Use a pacifier after delivery	87 (46.3%)

**Table 3:** Prevalence of outcomes 1 and 2.

**Did you stop breastfeeding?**	**Yes**			
	139 (76.8%)			
**How old was your infant when you totally stopped breastfeeding?**	Have not stopped	0–6 months	7–11 months	12 months or more
	42 (23.2%)	64 (35.4%)	12 (6.6%)	63 (34.8%)

**Table 4:** Association between sociodemographic and lifestyle variables and breastfeeding.

Variable	Did You Stop Breastfeeding?			*p*-Value		Infant Age When Breastfeeding Was Stopped			*p*-Value
		Yes	No		Have not Stopped	0–6 Months	7–11 Months	≥12 Months	
		*N* (%)	*N* (%)		*N* (%)	*N* (%)	*N* (%)	*N* (%)	
**Mother’s age**	21–25	26 (81.3%)	6 (18.8%)	0.644	6 (18.8%)	16 (50.0%)	1 (3.1%)	9 (28.1%)	0.370
	26–30	33 (70.2%)	14 (29.8%)		14 (29.8%)	14 (29.8%)	2 (4.3)	17 (36.2%)	
	31–35	46 (78%)	13 (22%)		13 (20.0%)	16 (27.1%)	7 (11.9%)	23 (39.0%)	
	36–40	34 (79.1%)	9 (20.9%)		9 (20.9%)	18 (41.9%)	2 (4.7%)	14 (32.6%)	
**Nationality**	Qatari	61 (82.4%)	13 (17.6%)	0.226	13 (17.6%)	31 (41.9%)	7 (9.5%)	23 (31.1%)	0.321
	Non-Qatari	77 (72.6%)	29 (27.4%)		29 (27.4)	33 (31.1%)	5 (4.7%)	39 (36.8%)	
**Number of children**	One	39 (79.6%)	10 (20.4%)	0.186	10 (20.4%)	24 (49.0%)	5 (10.2%)	10 (20.4%)	* 0.025
	Two	42 (84.0%)	8 (16.0%)		8 (16.0%)	17 (34.0%)	1 (2.0%)	24 (48.0%)	
	Three or more	58 (70.7%)	24 (29.3%)		24 (29.3%)	23 (28.0%)	6 (7.3%)	29 (35.4%)	
**Sex of last child**	Female	78 (80.4%)	19 (19.6%)	0.215	19 (19.6%)	43 (44.3%)	6 (6.2%)	29 (29.9%)	* 0.059
	Male	61 (72.6%)	23 (27.4%)		23 (27.4%)	21 (25.0%)	6 (7.1%)	34 (40.5%)	
**Knowledge**	Moderate	29 (80.6%)	7 (19.4%)	0.567	7 (19.4%)	15 (41.7%)	2 (5.6%)	12 (33.3%)	0.800
	Good	108 (76.1%)	34 (23.9%)		34 (23.9%)	47 (33.1%)	10 (7.0%)	51 (35.9%)	
**Attitude**	Negative	17 (89.5%)	2 (10.5%)	0.174	2 (10.5%)	8 (42.1%)	2 (10.5%)	7 (36.8%)	0.541
	Positive	121 (75.6%)	39 (24.4%)		39 (24.4%)	56 (35.0%)	10 (6.3%)	55 (34.4%)	
**Practice**	Bad	29 (87.9%)	4 (12.1%)	0.082	4 (12.1%)	21 (63.6%)	3 (9.1%)	5 (15.2%)	* 0.001
	Good	97 (73.5%)	35 (26.5%)		35 (26.5%)	37 (28.0%)	9 (6.8%)	51 (38.6%)	
**Did not know how to breastfeed**	Did not contribute to stopping	98 (75.4%)	32 (24.6%)	0.286	32 (24.6%)	37 (28.5)	9 (6.9%)	52 (40.0%)	* 0.022
	Contributed to stopping	39 (83.0%)	8 (17.0%)		8 (17.0%)	25 (53.2%)	3 (6.4%)	11 (23.4%)	
**Not making enough milk for baby’s needs**	Did not contribute to stopping	56 (75.7%)	18 (24.3%)	0.692	18 (24.3%)	17 (23.0%)	3 (4.1%)	36 (48.6%)	* 0.004
	Contributed to stopping	79 (78.2%)	22 (21.8%)		22 (21.8%)	44 (43.6%)	9 (8.9%)	26 (25.7%)	
**Had to return to work/school**	Did not contribute to stopping	68 (70.8%)	28 (29.2%)	* 0.022	28 (29.2%)	28 (29.2%)	6 (6.3%)	34 (35.4%)	0.084
	Contributed to stopping	65 (85.5%)	11 (14.5%)		11 (14.5%)	33 (43.4%)	6 (7.9%)	26 (34.2%)	

* *p*-value based on the Chi-squared test, *p*-value less than 0.05 is significant.

**Table 5:** Association between significant predictors and breastfeeding.

Variable	OR (95% CI)	*p*-Value
Number of children	0.806 (0.493–1.320)	0.392
Sex of last child	1.602 (0.741–3.464)	0.231
Practice	0.468 (0.141–1.546)	0.213
I did not know how to breastfeed	1.356 (0.535–3.435)	0.521
I was not making enough milk for my baby’s needs	0.782 (0.345–1.773)	0.556
I had to return to work/school	2.698 (1.162–6.267)	* 0.021

OR = odd ratio, CI = confidence interval, * *p*-value based on Chi-squared test, *p*-value less than 0.05 is significant.
